# 

**Published:** 1906-10

**Authors:** 


### ATLANTA JOURNAL-RECORD OF MEDICINE.

OCT 15

Successor to Atlanta Medical and Surgical Journal, Established 1855, and Southern Medical Record, Established 1870.

VOL. VIII. ATLANTA, GA., OCTOBER, 1906. NO. 7.

